# Kaposi Sarcoma, a Trifecta of Pathogenic Mechanisms

**DOI:** 10.3390/diagnostics12051242

**Published:** 2022-05-16

**Authors:** Gabriela Rusu-Zota, Oana Mădălina Manole, Cristina Galeș, Elena Porumb-Andrese, Otilia Obadă, Cezar Valentin Mocanu

**Affiliations:** 1 Department of Pharmacology, Clinical Pharmacology and Algesiology, Faculty of Medicine, University of Medicine and Pharmacy “Grigore T. Popa” Iasi, 700115 Iasi, Romania; rusu.i.gabriela@umfiasi.ro; 2 Faculty of Medicine, University of Medicine and Pharmacy “Grigore T. Popa” Iasi, 700115 Iasi, Romania; 3 Department of Histology, University of Medicine and Pharmacy “Grigore T. Popa” Iasi, 700115 Iasi, Romania; cgales81@yahoo.com; 4 Department of Dermatology, University of Medicine and Pharmacy “Grigore T. Popa” Iasi, 700115 Iasi, Romania; elena.andrese1@umfiasi.ro; 5 Department of Ophthalmology, University of Medicine and Pharmacy “Grigore T. Popa” Iasi, 700115 Iasi, Romania; obada_otilia@yahoo.com; 6 Department of Anatomical Pathology, University of Medicine and Pharmacy “Grigore T. Popa” Iasi, 700115 Iasi, Romania; mocanu.v.cezar@gmail.com

**Keywords:** Kaposi’s sarcoma, immunosuppression, human herpes virus 8, skin cancer, angiogenesis, oncogenesis, immune modulation

## Abstract

Kaposi’s sarcoma is a rare disease with four known variants: classic, epidemic, endemic and iatrogenic (transplant-related), all caused by an oncogenic virus named Human Herpes Virus 8. The viral infection in itself, along with the oncogenic properties of HHV8 and with immune system dysfunction, forms the grounds on which Kaposi’s Sarcoma may develop. Infection with HHV8 occurs through saliva via close contacts, blood, blood products, solid organ donation and, rarely, vertical transmission. Chronic inflammation and oncogenesis are promoted by a mix of viral genes that directly promote cell survival and transformation or interfere with the regular cell cycle and cell signaling (of particular note: LANA-1, v-IL6, vBCL-2, vIAP, vIRF3, vGPCR, gB, K1, K8.1, K15). The most common development sites for Kaposi’s sarcoma are the skin, mucocutaneous zones, lymph nodes and visceral organs, but it can also rarely appear in the musculoskeletal system, urinary system, endocrine organs, heart or eye. Histopathologically, spindle cell proliferation with slit-like vascular spaces, plasma cell and lymphocyte infiltrate are characteristic. The clinical presentation is heterogenic depending on the variant; some patients have indolent disease and others have aggressive disease. The treatment options include highly active antiretroviral therapy, surgery, radiation therapy, chemotherapy, and immunotherapy. A literature search was carried out using the MEDLINE/PubMed, SCOPUS and Google Scholar databases with a combination of keywords with the aim to provide critical, concise, and comprehensive insights into advances in the pathogenic mechanism of Kaposi’s sarcoma.

## 1. Introduction

Although it is a fairly rare disease, Kaposi’s sarcoma (KS) warrants attention due to its targeting of a vulnerable group, namely immunocompromised patients, through disease (human immunodeficiency virus (HIV)) or medication (iatrogenic). It was first described in 1872 by Moritz Kaposi in an article called Idiopathic multiple pigmented sarcoma of the skin, in which he presented five cases, each pertaining to men of older age and involving painful lesions of the skin, namely plaques and nodules. This would later be known as classic Kaposi’s sarcoma [1].

Later on, during the acquired immunodeficiency syndrome (AIDS) epidemic, a new variant of KS emerged, namely the AIDS-related variant [2], which distinguished itself for its aggressiveness. This is now known as epidemic KS. 

In Africa, the increased number of cases of KS would later come to define the endemic variant [3]. This variant, manifested with generalized lymphadenopathy, had increased significantly in the pediatric population [4].

The fourth and final variant of KS known is post transplantation and iatrogenic [3]. Recently, the number of case reports of the iatrogenic variant of KS has grown [5] in the context of long-term corticosteroid therapy [6] and biological therapies, including Abatacept administered for rheumatoid arthritis [7].

All four variants described involve Kaposi’s sarcoma-associated herpesvirus (KSHV), but by itself the virus is not enough to start the oncogenic process. The second common factor is immunosuppression, or a malfunctioning immune system. Currently KS is defined as a human herpes virus 8 (HHV8) associated angioproliferative disease. Kaposi’s sarcoma is a trifecta of pathogenic mechanisms and develops only when a mix of viral infection, oncogenesis and chronic inflammation occur [3].

In the current review, we summarize recent findings on the cellular and molecular mechanisms involved in development of KS based on a literature search using scientific databases including MEDLINE/PubMed, SCOPUS and Google Scholar. The search process comprised the following word combinations: “Kaposi’s sarcoma” or “Kaposi’s Sarcoma-Associated Herpesvirus” or ” Human Herpes virus 8” AND ”pathogenesis” or ”molecular biology” or ”immune modulation”. Studies between 2010 and April 2022 were selected. Additional references were obtained from the reference lists of the already included studies.

## 2. Replicative Cycle of HHV8

Around 12% of all cancers worldwide are caused by oncogenic viruses [3,8]. Among these, only KSHV and the human papillomavirus are an absolute requirement of oncogenesis for both their respectively determined cancers, and both are direct carcinogens [9]. KSHV, also known as the Human gammaherpesvirus 8 (HHV8), is a double-stranded DNA and a *Rhadinovirus,* the only one of the genus with human tropism. As previously outlined, HHV8 is found in all types of Kaposi’s sarcoma [10], and is needed for Kaposi’s sarcoma to appear, although the infection by itself is not enough. However, unlike other herpesviruses, which are widespread worldwide, KHSV has a heterogenous geographical distribution; prevalence of KSHV is low in most parts of the world, except Mediterranean countries (10%) and sub-Saharan Africa (50–95%) [9]. 

Infection occurs through saliva via close contact, blood, blood products, solid organ donation and, rarely, mother to fetus [11].

In 1994, Chang et al. first identified the KSHV in the lesions of an AIDS-KS patient by comparing affected and normal tissues by polymerase chain reaction (PCR) amplification [12].

Endothelial cells, dendritic cells, monocytes, B cells, epithelial cells and fibroblasts are all susceptible to infection by KSHV according to a study done by Bechtel et al. in 2003 [13] and, more recently it has been shown to also infect central nervous system cells in AIDS patients [14]. KS is considered a neoplastic vascular neoplasm, but some have also called it a “multifocal reactive hyperplasia of the vascular endothelium”, calling into question whether it is a true sarcoma.

KSHV has an enveloped icosahedral capsid with 162 capsomers and a total diameter of about 150–200 nm. The capsid is surrounded by the tegument and the virus envelope, which is studded with multiple glycoproteins such as gB, gH, gL, gM, gN, ORF4 and gpK8.1A, that are vital for the binding and entry into the host cell [10]. Through the binding of a variety of different KSHV glycoproteins to host cell receptors, viral entry is achieved. One of the more ubiquitous cell surface molecules, heparan sulfate, aids in the binding of a variety of host cells [15]. Depending on the cell type, KSHV uses different entry receptors. For the infection of endothelial cells, KSHV is presumed to bind to host cell surface receptors such as integrins α3β1, αVβ5 and αVβ3, the cystine-glutamate transporter xCT, heparan sulfate, and the tyrosine protein kinase receptor EPHA2 [16]. After binding, the KSHV enters cells via endocytosis [15,16].

Like many other viruses, KSHV has a lytic (short period) and latent (dormant—predominant for most of the time) phase [10]. 

### 2.1. Latency Phase

During its latent phase, KSHV does not cause its host to exhibit any obvious signs of pathology [10]. After viral entry into the host’s cell, the latent phase starts and a very limited number of genes are expressed during this phase. The corresponding proteins of these genes are latency-associated nuclear antigen (LANA), viral interferon regulatory factor 3 (vIRF3/LANA2), vCyclin, viral FLICE inhibitory protein (vFLIP), kaposin and viral miRNAs [17,18,19]. They, along with their effects, are presented in Table 1 [17,19,20,21,22,23,24,25,26,27,28,29,30,31,32,33].

### 2.2. Reactivation and Lytic Phase

The lytic reactivation represents another critical step in tumorigenesis, as demonstrated by the finding that inhibition of the lytic cycle using Ganciclovir reduced the risk of developing KS by 75% [34]. The lytic cycle is thought to provide signals that stimulate proliferation of latent cells, and thus of the tumor. This phase of viral infection represents the expression of viral proteins, replication of the genome, and assembly of new virions by the host cell, which exit the cell via budding. Stimuli that start off the lytic cycle are not well defined, but the process can be induced by substances such as 12-O-Tetradecanoyl-phorbol-13-acetate (TPA), sodium butyrate [35], ionomycin [36] (a calcium ionophore), epinephrine and norepinephrine at physiological concentrations, certain cellular factors (X-box binding protein 1 (XBP-1), CREB-binding protein (CBP), the SWI/SNF chromatin remodeling complex, the TRAP/Mediator complex, RBP-Jκ, human Notch intracellular domain, and High mobility group box 1 (HMGB1)) [37,38,39,40,41,42,43], autonomic nervous system activity within AIDS patients [44], hypoxia [45], and reactive oxygen species (ROS) [46]. Recently, it was also demonstrated that nitric oxide (NO) plays an important role in proliferation of KSHV associated tumors and is necessary for the lytic phase. According to Herrera-Ortíz et al., suppression of NO results in a reduced level of infectious virions, lytic transcripts and proteins [47]. SARS-CoV-2 viral proteins, namely the S and N proteins, have been noted to induce lytic reactivation of KSHV, thus accelerating the oncogenic process [48]. Moreover, in the same study, Chen et al. also reported that some anti-COVID-19 drugs used as of the date of their study, such as Azithromycin and Nafamostat mesylate, also contribute to the lytic reactivation of KSHV, and that AIDS-KS tissues have a higher ACE2 receptor expression, although a clear link between KSHV and the upregulation has not been established [48]. CD147, a multifunctional glycoprotein upregulated during KSHV de novo infection and in Kaposi sarcoma tissues [49,50] also represents one of the co-receptors for SARS-CoV2 entry into host cells [51]. Other viruses may also trigger the reactivation of KSHV lytic cycle, such as HIV, herpes simplex virus type 1 (HSV-1), HSV-2, human cytomegalovirus (HCMV), human herpesvirus-6 (HHV-6) and HHV-7 [52,53,54,55,56]. Spindle cells, with typical spindle-shaped morphology, which are the tumor cells of KS, tend to segregate the latent viral genomes and, as such, lytic reactivation of small populations of cells must occur to maintain viral presence and latency [57].

Genes that are expressed in the lytic cycle are divided into three groups: immediate early (IE), early (E) and late (L) genes. The lytic phase genes of the IE and E groups of most relevance to KS are summarized in Table 2. Late genes mostly comprise viral structural components [58].

## 3. Histogenesis

As previously mentioned, HHV8 is insufficient by itself to cause KS. A combination of a compromised immune system (whether achieved through medication or through a condition such as AIDS), genetic predisposition and environmental factors is needed for oncogenesis.

Most tumors present a single cell as a monoclonal point of origin; however, KS sometimes presents itself as oligoclonal [84,85]. The lesion, at the start at least, is considered reactive in nature (a reactive angioproliferative response towards HHV8), and only after the continued effects of the viral genes along with disruption of cell cycle arrest points, cytokine stimulation and local inflammation, can the KSHV-infected endothelial cells become a true neoplasm [84,85,86].

LANA-1, the major protein expressed during the latent phase, upregulates vFlip (which has antiapoptotic functions) and vCyclin (overcomes cell cycle arrest of RB through phosphorylation) [87], and stabilizes beta-catenin (through interaction with GSK-3beta) [88,89] and c-Myc (LANA decreases c-Myc phosphorylation thus inhibiting c-Myc induced apoptosis) [90], and also inhibits the p53 tumor suppressor gene. p53 is a known tumor suppressor [91,92] and its activity is commonly disrupted (via mutation or inhibition) in various cancers such as breast, lung, colon, esophageal, liver, brain cancers and sarcomas [93]. Similar to LANA-1, viral interferon regulatory factors (vIRFS 1 to 4) and the KSHV replication and transcription factor (RTA) also show anti-p53 activity [29,94,95]. LANA-1 thus contributes to the survival and immortalization of future tumor cells.

v-IL6, a protein expressed both during the latent (at lower but functional levels) and lytic viral phase of KSHV, plays an important role in KS genesis through angiogenesis, cell proliferation and invasion via downregulation of caveolin 1 (CAV1) [96], which has been shown to play an important role in survival, proliferation and migration of endothelial cells, as well as inhibiting pericyte and smooth muscle cell proliferation [97]. Downregulation of CAV1 occurs via v-IL6 signaling, which results in the formation of a STAT3-DNMT1 (DNA methyltransferase1) complex. This complex methylates the CAV1 promoter, thus leading to epigenetic silencing of said promoter. According to Li et al., deletion of the v-IL6 gene resulted in the abolishment of KSHV-induced cellular transformation and angiogenesis impairment [96]. Furthermore, Li et al. [96] indicated in their study that CAV1 might be a prospective target for future treatments of KSHV-associated cancers, including KS. 

vBCL-2 is encoded by the ORF16 gene and is a functional homologue of the human B-cell lymphoma 2 (BCL-2) gene [80]. vBCL-2 serves as both a way to extend cell survival via apoptosis inhibition and also evade the autophagic innate system [76,77,98]. Human BCL-2 is both a strong inhibitor of apoptosis and a potent proapoptotic factor when cleaved by caspases. KS vBCL-2 cannot be cleaved by caspases and thus cannot induce apoptosis, although it has been shown to have proapoptotic activity when expressed as an N-terminal truncation [98]. BCL-2 usually binds Beclin-1 (a proapoptotic protein) when cells are not starved. During cell starvation, the BCL-2/Beclin-1 complex is dissociated, and Beclin-1 induces autophagy [77,78]. vBCL-2, on the other hand, does not dissociate from Beclin-1 during starvation [78]. Thus, vBCL-2 promotes cell survival. 

vCyclin forms a complex with CDK6 which phosphorylates BCL-2. vCyclin accelerates cell-cycle progression and also promotes apoptosis in cells with increased levels of CDK6. This complex inactivates BCL-2 through phosphorylation; however, vBCL-2 escapes inactivation from vCyclin and continues to promote cell survival [99]. 

Another molecule that serves to inhibit apoptosis is the viral inhibitor of apoptosis protein (vIAP) encoded by the ORFK7 gene [100]. In a study done by Feng et al. [101] vIAP was shown to promote IκB and p53 tumor suppressor degradation via interaction with cellular PLIC1/ubiquilin, thus releasing inactive NF-kB, which promotes chronic inflammation. Activation of NF-kb also has a wide variety of effects. It promotes survival via upregulation of genes such as BCL-2-family members, FLIP a caspase regulator, cellular inhibitors of apoptosis c-IAP1, c-IAP, X chromosome linked IAP, and others [102]. NF-kb also protects cells from the effects of TNF-R mediated cytotoxicity via regulation of the JNK cascade. Removal of the NF-kb activity leads to programmed cell death [102].

K1 plays an important role in lytic replication, cellular survival and immortalization of human endothelial cells [103]. Through its cytoplasmic tail, K1 was able to activate the PI3K pathway, and consequently the Akt kinase and mTOR, and inactivate proapoptotic proteins FKHR, glycogen synthase kinase-3 (GSK3) and Bad (thus promoting cell survival) [103,104]. K1 also enhances angiogenesis and increases tumor size [103].

Physiologically, angiogenesis represents the formation of new blood vessels from existing ones. The migration, growth and differentiation of endothelial cells are involved in this process. Many tumors require angiogenesis for growth. Tumors often stimulate angiogenesis to supply themselves with nutrients and to sustain themselves [105]. Those that grow too fast or fail to obtain the necessary blood supply necrose from center to periphery; in these cases, there is true hypoxia. Both fast and slow growing tumors eventually create a microenvironment that stimulates the hypoxia-inducible factor (HIF-1) and consequently VEGF-A [106]. 

Kaposi’s sarcoma development is somewhat different (but not unique). It can promote VEGF-A secretion in normoxic conditions by several methods by way of KSHV proteins. KSHV genes that contribute to the stabilization of HIF-1 (which is normally degraded in normoxia [106]), include LANA-1 [107,108], vIRF3 [109] and viral G-protein coupled receptor (vGPCR) [110]. As noted by Carroll et al. [111] latently infected cells increase HIF-1 transcription but do not necessarily stabilize it, whereas vGPCR stabilizes it via posttranslational modification [111]. 

Direct secretion of VEGF-A is promoted by viral products such as glycoprotein b (gB) [112,113], K1 [103], K8.1 [112,113], vIL-6 [69] and miRNAs (via downregulation of THBS1). 

Although not a direct promoter of angiogenesis via VEGF-A, K15 has been shown to induce angiogenic tube formation via a phospholipase Cγ1 (PLCγ1) VEGF-dependent pathway, even when the VEGF receptors were silenced [72]. K15 has also been shown to increase proliferation and migration of endothelial cells via store-operated calcium entry [73]. 

Another indirect promoter of angiogenesis is the KSHV miRNAs, which have been shown to target and downregulate THBS1, an inhibitor of angiogenesis that regulates VEGF and a potent activator of latent TGF-beta, both free and cell-associated [24,114,115,116,117,118]. Tat, a protein that was previously mentioned as playing an important role in AIDS-associated KS, is also inhibited by THBS1 [114]. 

VEGF induces activation of RAC1 [119] (and consequently p21-activated kinase (PAK1)) which increases vascular permeability [120]. These changes are found in latently infected endothelial cells, as shown by Guilluy and colleagues [121]. RAC1 is a known member of the Rho family of GTPases as well a regulator of multiple pathways (e.g., cell proliferation, transformation, migration) [122]. P21-activated kinase plays a role in regulating endothelial permeability as a downstream regulator of Rac1 [120]. 

KSHV infected cells also demonstrate an increased endothelial permeability due to the downregulation and degradation of VE-cadherin [123]. Cadherins types of cell adhesion molecules that cells use to form adherens junctions between each other. Vascular endothelial cadherin (VE-cadherin) plays an important role in maintaining endothelial cell cohesion. K5 has been shown by Mansouri et al. to promote downregulation of VE-cadherin [70] and platelet-endothelial PECAM/CD31 (targeting both existing—via ubiquination—and newly synthesized CD31 via endoplasmic reticulum proteasomes) [124]. P120 has been shown to have a protective effect for VE-cadherin from KSHV K5; however, K5 still displaces it and degrades the endothelial junctions as a result [125]. A study by Dwyer et al. [126] also indicates that vGPCR expression, via the PL(3)Kγ/Rac pathway, leads to the loss of endothelial cell junctions and increase in permeability by hyperphosphorylation of VE-cadherin [127].

Angiopoietin 2 is increased in KS lesions [128]. vGPCR, along with v-IL6, also upregulates the expression of angiopoietin-2 in infected endothelial cells, an antagonist of the angiopoietin receptor Tie2. This increased expression promotes angiogenesis through the ERK, JNK, p38 mitogen-activated protein kinase (MAPK) pathways via AP-1 and Ets1 activation [129]. As pointed by out by Ye et al. [129], infection of HUVEC cells with KSHV induced expression and release of angiopoetin-2 along with formation of spindle cells reminiscent of KS tumors. 

Angiopoietin-like 4, a molecule that promotes angiogenesis and vascular permeability, is also upregulated by vGPCR and has a higher expression rate in oral KS lesions than VEGF [130]. vGPCR and vIL-6 also promote angiogenic response through viral CC chemokine ligands in PEL cell cultures [131,132].

The precursor for the KS spindle cell is yet to be determined with certainty. However, we know, according to studies done in the past, that spindle cells present both lymphatic endothelial cell (LEC) markers of differentiation as well as blood endothelial cell markers (BEC). The prevailing idea is that BEC are reprogrammed to LEC [133,134]. KSHV-infected BEC have been shown to express genes similar to LEC, such as prox-1, VEGFR3 (VEGF receptor 3), podoplanin and LYVE-1 (Lymphatic vessel endothelial receptor 1) [133,134,135]. Reprogramming of BEC towards an LEC type allows the KSHV infected cells to respond to both VEGF-A and VEGF-C (which promotes lymph angiogenesis) [113]. VEGF-C serves a role similar to VEGF-A, inducing migration and proliferation of KS spindle cells [136]. Viral mi-RNAs, miR-K6 and miR-K11 have been shown to redirect BEC to LEC via the musculoaponeurotic fibrosarcoma oncogene homolog downregulation [22].

Matrix metalloproteinases are endopeptidases whose main function is extracellular matrix (ECM) remodeling in physiological conditions. In a study done by Pantanowitz et al. [137], it was shown that KS lesions are immunoreactive for almost all matrix metalloproteinases (MMPs) included in the study, such as collagenases (MMP-1, MMP-13), gelatinases (MMP-2. MMP-9), stromelysin-1 (MMP-3—only in tumor cells), and matrilysin (MMP-7), membrane type MMP-14 (only in regressed lesions). In KS, MMPs serve to increase angiogenesis through basement membrane disruption and other ECM barriers, allowing invasion and migration of endothelial and Kaposi spindle cells (KSC) [138]. In a study done by Bongiorno et al. [138], MMP-2 and MMP-9 were shown to have similar immunoreactivity profiles in AIDS-associated KS vs. classic KS lesions. However, in AIDS patients, the tat (Trans-Activator of Transcription) protein encoded by the human immunodeficiency virus 1 increases MMP-2 expression in AIDS-KS lesions and MMP-2 plasma levels, suggesting that tat could play a role in the higher aggressiveness of KS in AIDS patients [139]. Tetracycline derivatives can inhibit both enzymatic activity and synthesis of MMPs by blocking gene transcription. These derivatives can serve as MMP inhibitors; of this group, metastat (COL-3), has been noted as showing promise in treating epidemic KS [140].

Certain inflammatory cytokines are upregulated and others are downregulated as the result of viral infection [141]. vIRFs work along with LANA and k-bZIP to downregulate the IFN pathway. vIRF-1 interferes with the formation of the CBP/p300 complex and p300 HAT activity to downregulate IFN-α. vIRF-2 downregulates interferon-α (IFN-α), IFN-β and IFN-λ expression [142]. vIRF-3 suppresses IFN-α by binding to the DNA domain or the central IRF association domain of IRF7 [143]. LANA1 downregulates IFN-β expression via competitive binding with IRF3 to the IFB-β promoter [144]. K-bZIP/ORFK8, in turn, interacts directly with the IFN-β promoter to impede its binding with IRF3 [145]. 

KSHV additionally encodes three chemokine homologs known as viral CC-chemokine ligand 1 (vCCL1; encoded by K6), vCCL2 (K4) and vCCL3 (K4.1). These were previously known as viral macrophage inflammatory proteins (vMIPs). vCCL1 and vCCL attract Th2 and CD4 + CD25+ regulatory T cells that, in turn, serve to downregulate the immune response [146,147,148,149]. vCCL2, on the other hand, antagonizes Th1 cells via CCR1, CCR2, CCR5 and CX3CR1 and CXCR4. Moreover, vCCL2 impairs arrest of monocytes and Th1 cells, but promotes arrest of eosinophils and Th2 cells [150,151]. By infecting endothelial cells, KSHV induces expression of a variety of cytokines, including angiopoietin-2, IL-6, IL-10 and IL-13. These cytokines stimulate monocytes to differentiate into tumor-associated macrophages that promote angiogenesis and suppress T-cell responses [152,153,154]. Mast cells have been shown to be viral reservoirs and, moreover, secrete histamine, which stimulates KSHV viral replication [155,156]. B lymphocytes along with macrophages have also been shown to be viral reservoirs and, moreover, their circulation could spread the infection to other tissues [157,158].

But why does KS occur predominantly in men? A recent study conducted by Ding et al. [159], shows that androgen receptors (AR) play an important role in oncogenesis by facilitating lytic replication. Moreover, male steroid treatment leads to extensive lytic gene expression in infected cells and impaired expression of PAN RNA [159]. 

In conclusion, KS is the result of a “perfect storm” comprised of viral infection, chronic inflammation (B lymphocytes, plasmocytes, monocytes), and oncogenesis (proliferation, immortalization).

## 4. Clinical Presentation

KS, as previously mentioned, can be split into four main clinical-epidemiological types:

- Classic KS
- Endemic KS
- Epidemic KS
- Iatrogenic (post-transplant) KS

KS has a heterogenic clinical presentation; some patients have indolent disease and others have an aggressive disease.

Classic or sporadic KS is specific to older men of Mediterranean descent or Ashkenazi jews [160,161]. It typically occurs over the age of 60 at the lower limbs, is indolent, and cutaneous lesions are the most common form of presentation for this form. Visceral involvement is rare. Cigarette smoking has a protective effect [162]. 

Endemic KS is specific to Africa, where KSHV seroprevalence is over 40–50% [22,163]. Two subcategories of endemic have KS emerged: one specific to young children characterized by lymphadenopathy, visceral involvement and aggressive progression [164], and one that arises in males over the age of 40, which manifests indolently, frequently at lower limb level. Additional external factors, such as AIDS spread and fine soil particles associated with bare foot walking, may contribute to development of KSHV-associated lesions like KS [53,165]. 

Epidemic or AIDS-related KS describes AIDS-associated tumors. KS is the second most common tumor after non-Hodgkin’s lymphoma. This variant is extremely aggressive, and infection can predate KS by 10 years [166]. CD4 T-cell decline is a risk factor for developing AIDS-related KS [19].

Iatrogenic or post-transplant KS occurs when a patient receives immunosuppression that causes the reactivation of a previously HHV8 infection, or when the patient received an infected organ [167]. Interruption of immunosuppression, when possible, causes the regression of post-transplant KS. 

A new entity has been described in several case reports and named non-epidemic KS [168,169]. Moreover, in a cohort study of classic KS, Denis et al. [169] identified this fifth type of KS in a low-endemic zone for KSHV. This type of KS occurs in men who have sex with men and involves young or middle-aged patients without HIV infection. Similar to AIDS-KS, there was a correlation between the CD4 T-cell count and CD4/CD8 ratio at baseline and the severity of the disease. This type has few lesions, rare visceral involvement and is indolent [19].

The clinical types of KS have no homogenous staging. In classic KS, clinical manifestation with patch/papules, plaque and tumor limited to the lower extremities describes the first three stages of disease. Stage 4 implies disseminated diseases. In AIDS-related KS, the immune status and systemic illness, along with tumor location, are criteria for staging. Endemic and post-transplant KS have no specific staging [19]. 

In regard to the location where KS most commonly appears, mucocutaneous sites are most frequently seen. The cutaneous lesions are either flat or elevated, painless, pink to violaceous coloured and do not blanch under pressure [19]. Lymph nodes and visceral organs are also common locations. In a review, Pantanowitz et al. [170] identified unusual locations for KS and emphasized these as they can prove difficult to diagnose. The musculoskeletal system, urinary system, endocrine organs, heart and eye are considered among atypical sites for KS. In musculoskeletal KS, the ulcerating lesion engages the underlying bone with an osteolytic effect [170]. In ocular KS, lesions can be observed most commonly on the eyelids and conjunctiva and rarely can extend into the orbit, involve the lacrimal gland [171] or the nasolacrimal duct [172]. Conjunctival KS appears as bright red or violaceous flat lesions or a nodular, mobile mass involving the lower conjunctival fornix, the bulbar or the upper conjunctiva [173] that can mimic a conjunctival hemorrhage. In disseminated KS associated with AIDS, bilateral choroidal involvement has been found on autopsy [174]. The literature also cites cases of ocular KS with eyelid and conjunctival involvement as the initial manifestation of HIV-AIDS [175]. Central nervous system localization of KS is very rare and is related to HIV infection and disseminated KS [170,176].

## 5. Histopathology

All four variants of KS show similar histological features, and there is no clear difference between them. The three previously mentioned clinical stages, namely patch/papules, plaque and the tumor stage are not necessary for microscopic diagnostic but do help in recognizing the various patterns of KS. The most defining feature of KS is spindle cell proliferation, with differing forms of sieve and slit-like spaces occupied by erythrocytes and hemosiderin deposits, with a plasma cell and lymphocyte infiltrate. Sometimes, the erythrocytes might be missing, and thus hemosiderin deposits should be looked at as proof of extravasation of the previously mentioned cells.

The patch stage represents the earliest stage of KS. It is characterized by a subtle endothelial cell proliferation with minimal atypia, scant plasma cell and lymphocyte proliferate. The “promontory sign” can also be seen during this stage, which is characterized by other more mature structures (such as another vessel) protruding into the newly formed vessels (e.g., a “vessel-in-vessel” aspect), which also tend to wrap/and or dissect collagen bundles [177]. The newly formed vessels are leaky, easily permitting erythrocytes an exit. Hemosiderin deposits are indicative of the presence of erythrocytes when they themselves are not observable. 

Plaque stage is characterized by a more extensive vascular proliferation and plasma cell inflammatory infiltrate. Eccrine glands, if they are present on the slide, are sometimes useful for noticing an invasive process. Spindle cells will often be seen invading the eccrine glands or destroying them outright.

The tumor stage is the final and most representative stage of spindle cell proliferation—it is also called “nodular” and the neoplastic cells form solid aggregates of intersected spindle cells dotted by slit and sieve-like spaces with erythrocytes inside. The proliferation is so extensive, that the entire dermal layer up to the epidermis may be occupied by the spindle cell proliferate. Certain periodic acid-Schiff (PAS) positive hyaline globules are found within neoplastic endothelial cells and are believed to be phagocytized erythrocytes [178]. 

During early stages, using simple H&E stains, KS might mimic benign vascular proliferations, and during later stages might be confused with other malignant spindle cell tumors [179]. 

As ancillary tests, the following immunohistochemical stains are used: CD34 (+), CD31 (diffuse, weak + [180], can be explained by the increased degradation of PECAM [140]), ERG (+), D2-40/podoplanin (+), PROX1 (+), FLI1 (+) and nuclear HHV8 (LANA 1—which is essentially almost always positive in KS and negative in other proliferations of a vascular nature) [181].

## 6. Current Treatments and Experimental

There are different treatment options for patients with KS, classified as standard and targeted therapy. Standard treatment of KS include HAART (highly active antiretroviral therapy), surgery with local excision, cryosurgery, radiation therapy, chemotherapy and immunotherapy. Targeted therapy is a novel type of treatment that affects specific cells, thus causing less damage to normal cells. New therapies target the signals transduction pathways utilized by KSHV, such as inhibition of XPO1 [182] or KSHV thymidine kinase (TK/pORF21) [183,184] or MMPs, or HSP90 and HSP70 chaperons [183] or downstream of vGPCR [183,185].

Surgical excision is appropriate for small and superficial lesions but has the major drawback of high recurrence rate. Cryosurgery has temporary effects on superficial lesions and induces hypopigmentation [186]. 

AIDS-related KS is usually managed by ensuring an increase in CD4+ cells via HAART, which can lead to the regression of the tumor and improve the prognosis [6]. Chemotherapy can also be instituted if HAART by itself is not enough; therapeutic agents such as liposomal anthracyclines are used (e.g., pegylated liposomal doxorubicin (PLD)) [187]. Gleevec (Imatinib) has also been reported as being effective in some cases [188,189].

After initiating HAART in patients with immunosuppression there is a risk of KS immune reconstitution inflammatory syndrome (IRIS). IRIS is an inflammatory disorder associated either with a clinical paradoxical worsening of preexisting KS or, rarely, with unmasking an undiagnosed KS. IRIS management is not standardized. Dependent on clinical evolution, continuing HAART and adding chemotherapy are both options for managing IRIS in KS. Glucocorticoids should be avoided as they may worsen KS [19,190].

Classical KS can be treated with radiation therapy and chemotherapy (local or systemic) [191,192] with a generally favorable response. No vaccine and no antiviral agents are currently used against KS [10].

Iatrogenic KS is usually treated by removal of immunosuppressive therapy. If such actions are not possible, radiation therapy can be used instead [10]. Topical timolol has been noted as effective in one case of iatrogenic KS [193].

Immunotherapy is considered a salvage therapy and involves administration of monoclonal antibodies to block immune checkpoints in refractory KS to other treatments [194]. Although there are several series treated with Ipilimumab (anti-CTLA-4 antibody), Nivolumab and Pembrolizumab (anti-PD-1 antibodies) and Atezolizumab (anti-PD-L1 antibody), anti-immune checkpoints are still under evaluation and are not approved.

In a recent study, Meng et al. [182] showed that XPO1 (exportin 1) plays a crucial role in the KSHV lytic cycle during primary infection—inhibition of XPO1 induces p62 nuclear retention which activates TBK1 and IRF3 and enhances the expression of innate immunity related genes, thus blocking KSHV lytic replication (infectious virions and viral gene expression). XPO1 was inhibited using Eltanexor (KPT-8602). Eltanexor (KPT-8602), a Second-Generation Selective Inhibitor of Nuclear Export (SINE) compound, in the same study, was pointed out by Meng et al. as a potential therapeutic agent against KSHV infection. In 2019, Gruffaz et al. [195] showed that Eltanexor blocks cell proliferation and growth transformation by KSHV transformed cells by p53 mediated cell cycle arrest.

K15 plays an essential role in lytic replication and, as such, may constitute a potential therapeutic target as noted by Abere et al. [196]. Tyrosine kinase inhibition of ORF21/thymidine kinase was also shown to be a potential therapy by Beauclair et al. [184].

Sulfonamide compounds could possibly contribute in clearing latent KSHV, thus inhibiting pathogenesis and, thereby, oncogenesis [197]. 

## 7. Conclusions

Although remarkable progress has been made in understanding the pathogenesis of KSHV, extensive research is needed to understand the exact molecular mechanisms that are necessary for KS to appear. As previously outlined, infection by itself is not enough; the oncogenic properties of HHV8 and the immune system dysfunction are also needed for KS development. Therefore, any advance for a better understanding of KS pathogenesis provides hope for the discovery of new targeted therapeutics.

## Figures and Tables

**Table 1:** List of latent cycle expressed genes and their respective proteins.

Latent Cycle Gene—Protein	Function
ORF73—LANA	Serves as a means to circularize and attach the viral genome to the host’s chromosomes, but also inhibits p53 activity, tumor suppressor Rb, leads to progrowth proteins cyclin D and c-Myc upregulation and also extension of host cell life via telomerase expression.
ORF72—vCyclin	Homologue of cellular Cyclin D. Can bind and activate the cyclin-dependent kinase cdk6 and through this complex lead to the inactivation of tumor suppressor retinoblastoma (Rb), cdk inhibitor p27 (Kip), and the antiapoptotic protein Bcl-2.
ORF71/K13—vFlip	Is a homologue of caspase-8 inhibitory protein and has been shown to prevent the CD95 death receptor and cleavage of procaspase 8 (thus stopping the forming of active caspase 8).
ORFK12—Kaposins A, B and C	Kaposins A, B and C; Kaposin A plays a role in cellular transformation and activation of the ERK/MAPK pathway. Kaposin B binds and activates the p38/MAPK target kinase MK2 inhibiting the decay of mRNAs such as those for PROX1, thus inducing the reprogramming of endothelial cells towards a lymphatic lineage
miRNAs **	Promotes cell survival via apoptosis inhibition, and continuation of latent phase, endothelial cell reprogramming, induction of migration and invasion (via miR-K12–3). miR-Ul112 downregulates MICB expression and reduces infected cell killing by natural killer cells; suppression of thrombospondin 1 (THBS1), a known tumor suppressor, leads to lowered TGB-β and subsequently leads to a loss of anti-angiogenic activity, contributing to carcinogenesis. miRNAs are present in all KSHV associated diseases (KS, Multicentric Castleman’s Disease (MCD) and primary effusion lymphoma (PEL), body cavity based cell lymphoma)
ORFK10.5—vIRF3/LANA2	Specific to B-cells. Inhibits p53 tumor suppressor. Expressed uniformly in PEL tumor cells. LANA2 inhibits cell cycle arrest mediated by 14-3-3σ overexpression.

ORF = open reading frame, named based on the homologous genes in *Herpesvirus saimiri*. K genes are unique to KSHV. ** miRNAs share the same gene locus as kaposins; however, they are transcribed as independent genes.

**Table 2:** Major genes expressed during lytic cycle and their functions.

Gene—Protein	Function
ORF45—ORF45	(IE) Inhibits p53 signaling and prevents interaction with USP7 (a deubiquitinase), which results in diminished transcriptional activity [59].
ORFK4.2—ORFK4.2	(IE) Plays a role in immune evasion, lowering antibody-mediated adaptive immune responses [60].
ORFK12—Kaposins	(E) Kaposin B has been shown to contribute to angiogenesis, reprogramming of endothelial cells, which has a proinflammatory effect via citokine upregulation [61,62,63,64,65].
ORF57—ORF57	(E) Interacts directly with PYM to facilitate the efficient translation of intronless KSHV mRNA transcripts [66]. Protects viral products such as viral interleukin-6 (vIL-6) and IL-6 from miRNA degradation [67].
K-bZIP (ORF-K8)	(E) Modulator of RTA activity. Inhibits RTA autoactivation and transactivation of ORF57 and ORF-K15 [68].
K2—vIL-6	(E) Increased vascular endothelial growth factor a (VEGF-a) secretion (angiogenesis), tumor growth and plasmocytosis in mice [69].
K5—ubiquitin E3 ligases	(E) Disruption of endothelial cell adhesion via cadherin downregulation [70].
K14—vOX-2	(E) Stimulates productions of inflammatory cytokines and chemokines, such as IL-1β, IL-6, tumor necrosis factor α (ΤNF-α), and monocyte chemoattractant protein-1 (MCP-1) [71].
K15—K15	(E) Vascular endothelial growth factor receptor (VEGFR) independent angiogenesis stimulation [72]. Stimulates endothelial cell proliferation and migration [73].
ORF16—vBcl2	(E) Essential to KSHV replication [74,75]. Anti-apoptotic and anti-autophagy evasion functions [76,77,78,79,80].
K1—K1	(E) Activation of the Phosphoinositide 3-kinase (PI3K)/AKT/mammalian target of rapamycin (mTOR) pathway, which leads to upregulation of protein synthesis and survival, while also inhibiting apoptotic signaling [81].
ORF74—vGPCR	(E) Transformative properties [81]. Expression of vGPCR leads to immortalization of endothelial via VEGF receptor-2/KDR (kinase insert domain receptor) [82]. Knockdown of vGPCR is documented to lead to decreased tumor growth and lower secretion of VEGF in a mouse model [83].

## Data Availability

Not applicable.
